# What’s the “secret sauce”? How implementation variation affects the success of colorectal cancer screening outreach

**DOI:** 10.1186/s43058-020-00104-7

**Published:** 2021-01-11

**Authors:** Jennifer Coury, Edward J. Miech, Patricia Styer, Amanda F. Petrik, Kelly E. Coates, Beverly B. Green, Laura-Mae Baldwin, Jean A. Shapiro, Gloria D. Coronado

**Affiliations:** 1 Oregon Rural Practice-based Research Network, Oregon Health & Science University, 3181 S.W. Sam Jackson Park Rd., Mail Code L222, Portland, OR 97239 USA; 2 Center for Health Services Research, Regenstrief Institute, Indianapolis, IN USA; 3 Business Administration, Southern Oregon University, Ashland, OR USA; 4 Center for Health Research, Kaiser Permanente Northwest, Portland, OR USA; 5 Quality Improvement Program Administrator, CareOregon, Inc., Portland, OR USA; 6 Kaiser Permanente Washington Health Research Institute, Kaiser Permanente Washington, Seattle, WA USA; 7 Department of Family Medicine, University of Washington School of Medicine, Seattle, WA USA; 8 Division of Cancer Prevention and Control, Centers for Disease Control and Prevention, Chamblee, GA USA

**Keywords:** Implementation, Colorectal cancer, Program adaptation, Cancer screening outreach, Cancer prevention

## Abstract

**Background:**

Mailed fecal immunochemical testing (FIT) programs can improve colorectal cancer (CRC) screening rates, but health systems vary how they implement (i.e., adapt) these programs for their organizations. A health insurance plan implemented a mailed FIT program (named BeneFIT), and participating health systems could adapt the program. This multi-method study explored which program adaptations might have resulted in higher screening rates.

**Methods:**

First, we conducted a descriptive analysis of CRC screening rates by key health system characteristics and program adaptations. Second, we generated an overall model by fitting a weighted regression line to our data. Third, we applied Configurational Comparative Methods (CCMs) to determine how combinations of conditions were linked to higher screening rates. The main outcome measure was CRC screening rates.

**Results:**

Seventeen health systems took part in at least 1 year of BeneFIT. The overall screening completion rate was 20% (4–28%) in year 1 and 25% (12–35%) in year 2 of the program. Health systems that used two or more adaptations had higher screening rates, and no single adaptation clearly led to higher screening rates. In year 1, small systems, with just one clinic, that used phone reminders (*n* = 2) met the implementation success threshold (≥ 19% screening rate) while systems with > 1 clinic were successful when offering a patient incentive (*n* = 4), scrubbing mailing lists (*n* = 4), or allowing mailed FIT returns with no other adaptations (*n* = 1). In year 2, larger systems with 2–4 clinics were successful with a phone reminder (*n* = 4) or a patient incentive (*n* = 3). Of the 10 systems that implemented BeneFIT in both years, seven improved their CRC screening rates in year 2.

**Conclusions:**

Health systems can choose among many adaptations and successfully implement a health plan’s mailed FIT program. Different combinations of adaptations led to success with health system size emerging as an important contextual factor.

Contributions to the literature

- Our article analyzes adaptations that enable health care staff to implement mailed fecal immunochemical testing (FIT) programs in delivery systems.
- Our results explore which adaptations made by health systems to mailed FIT programs are related to screening rate improvements. Our analysis shows that a health system’s organizational characteristics in combination with specific adaptations linked directly to CRC screening rates and implementation outcomes.
- Our article describes different pathways that health systems can use to implement CRC screening outreach to improve colorectal cancer screening participation. These pathways show that implementation flexibility and customizing colorectal cancer screening outreach to particular clinic contexts can enhance implementation success.

## Background

Colorectal cancer (CRC) screening remains an underutilized preventive health measure despite its effectiveness at reducing mortality and morbidity [1, 2]. Only 60% of US commercially or Medicare-insured adults are up-to-date on screening, and the rate is even lower for Medicaid-insured adults (47%) [3]. Numerous US state and federal programs, health care systems, and insurance plans are trying to improve rates of CRC screening using various population-based approaches [4, 5]. The most successful organizations apply multifaceted, population-based strategies [6].

Some health care systems are working to raise CRC screening rates through screening outreach programs, such as those that mail fecal immunochemical test (FIT) kits to patients due for screening [7–9]. Clinic- and health care system-based outreach efforts have increased screening rates, and studies have demonstrated that more screening is associated with reduced CRC incidence and cancer mortality [10–15]. Despite the effectiveness of screening outreach, and specifically for mailed FIT programs [16, 17], challenges remain with implementation of such programs in practice [7, 18, 19].

One promising approach that addresses some barriers faced by health systems is mailed FIT outreach initiated by health insurance plans [20]. Health plan-initiated mailed FIT programs can minimize the burden on clinics, and lower program costs by creating efficient ways to implement the programs [6, 20]. Prior research shows that an evidence-based program of CRC outreach in health systems and clinics is typically adapted to fit an organization’s structure and available resources [21–25]. Qualitative findings from this approach have indicated a mailed FIT program can be adapted to the culture and needs of individual health insurance plans [18, 26]. Little information exists, however, on how health systems adapt their programs over time, and *which adaptations* are most effective for positive outcomes.

To address some of these questions, a collaboration (named BeneFIT) was formed between researchers and health insurance plans to understand the implementation of a health plan-driven mailed FIT program. A health plan in Oregon coordinated and administered the mailing of FIT kits while partnering with the health systems that delivered care to their health plan members. The research team has previously reported on the BeneFIT program’s effectiveness using a research sample from six health systems and found that of those who were mailed an introductory letter, FIT, and reminder postcard, 18.3% completed the FIT, and 20.6% completed any CRC screening [27]. However, FIT completion rates varied greatly (from 10.0 to 21.1%) across the health systems in that research study [27]. A similar mailed FIT intervention in a pragmatic trial (STOP CRC) also showed improved screening rates with substantial variation between health systems [28].

The Oregon BeneFIT mailed outreach program used a collaborative model, in which health systems were able to customize the basic mailing offered by the health plan and choose options for how to implement the program in a way that corresponded with health system organizational constraints and capabilities. This paper examines the major adaptations made to the mailed FIT program during implementation in relation to CRC screening rates. We then identify which combinations of adaptations uniquely distinguished health systems with higher CRC screening rates.

## Methods

### Setting

The health insurance plan is a non-profit organization that provides insurance in Oregon for Medicaid, Medicare, and dental coverage for about 220,000 enrollees at the time of the study. The mailed FIT program was implemented in collaboration with a research team in 2016 (May to November) and 2017 (May to November). A total of 17 health systems took part in the BeneFIT program offered by the Oregon health insurance plan over the first 2 years that the program was implemented. We report here on 2-year CRC screening rates and implementation variations (i.e., adaptations) for these health systems. Six of these health systems had the capacity to provide FIT test results to the research team and implement the program quickly enough to be included in a prior analysis of the 1-year mailed FIT outcomes [27].

### Mailed FIT intervention

The BeneFIT program is described in detail elsewhere [20]. Briefly, health plan staff generated lists of enrollees due for CRC screening for each health system that took part in the program. To be eligible for the mailed FIT program, a member must have been between the ages of 51 and 75 and not have had a health plan claim indicating CRC screening or a screening exclusion (i.e., colon cancer). Health plan staff provided the member lists and FIT kits to a mail vendor that prepared and mailed introductory letters. Enrollees whose introductory letter was returned as undeliverable were removed from the list. The mail vendor mailed remaining enrollees a FIT kit about 4 weeks later, followed by a postcard reminder 2 weeks later.

Within this framework, each health system was allowed by the health plan to customize how they implemented the program. For all health systems, the FIT results came back directly to clinics and were followed up directly by the patient care teams using the clinics’ usual care procedures. The basic program (specifically the mailing elements coordinated by the health plan) was presented to clinic managers, who then determined if they would be able to add clinic-supported adaptations, such as phone call reminders. The adaptations (i.e., differences in implementation) fell into five types:

- *Lists of eligible enrollees scrubbed before mailing the introduction letters*: Health systems could review the list of eligible members that the health plan generated and remove patients based on their own patient data. Health system staff either looked for patients who were current for screening according to clinic-based medical records or simply validated that the patients correctly belonged to the clinic’s population [e.g., were regularly seeing one of their providers or had an electronic health record (EHR)]. The health system then returned a “scrubbed” list back to the health plan.
- *Twelve-month visit exclusion*: Some clinics chose to have the health plan automatically exclude patients who had not had a clinic visit in the last year. In this case, the health plan staff removed patients without a visit in the last 12 months using the claims database. (Often, this adaptation was chosen simply because clinics could not staff the effort of scrubbing the mailing lists.)
- *Phone call reminders*: Some health systems had staff call patients who were mailed an introduction letter and FIT kit to remind them to return the test. The health plan provided the clinic staff with a list of plan members who were mailed an introduction letter and FIT kit.
- *Financial incentives (gift card) offered for completing CRC screening*: In some regions or health systems, the health plan offered incentives for completion of CRC screening (either by FIT or by colonoscopy). The incentives ($25 gift cards) were mentioned in the letters that accompanied the FIT kits.
- *Allowing FIT kits to be mailed back (vs. requiring in-person drop off)*: Some health systems required members to return the completed FIT kits in person to a clinic. Other health systems allowed members to mail back the completed kits in pre-stamped return mailers that were provided when the kits were sent (referred to as a mailed return).

In addition to these five major implementation variations, other health care system characteristics were available for the analysis. Some of the health systems had participated in prior research efforts involving mailed FIT outreach and therefore had some existing FIT mailing workflows and staff experience. The health systems varied in size, both in number of clinics and number of patients they served. Finally, the health plan allowed the program to mail whichever type of FIT was already in use by the health system. All health systems used one of the following three types of FIT: the two-sample Insure® by Clinical Genomics, one-sample Hemosure® by Hemosure, Inc., or one-sample OC-Light® or OC-Auto® by Polymedco.

### Study measures

The main outcome for these analyses was completed CRC screening rates. A screening was considered complete if a claim was submitted that indicated a patient received any type of CRC screening procedure within 6 months of the date that the introductory letter was mailed. A CRC screening procedure was defined as any of the following:

- FIT test or fecal occult blood test (FOBT)
- FIT-DNA test
- Flexible sigmoidoscopy
- Computed tomography (CT) colonography (virtual colonoscopy)
- Colonoscopy.

The number of FIT kits mailed indicates the number of eligible health plan members who were mailed a FIT kit through the BeneFIT program. All implementation outcomes were tracked internally by health plan staff as they generated lists of eligible patients and worked with health systems and the mailing vendor to conduct the mailing itself [27]. For FIT kits mailed in late 2017, there was a minimum 3-month period for claims to be received by the health plan following the 6-month screening period.

Each variable was a potential explanatory factor that could have a plausible connection to the outcome. Health plan characteristic variables included health system name, health system size (number of clinics per system), participation in the prior CRC screening study, and FIT test type used by the health system. Intervention variables included the length of participation in BeneFIT, number of adaptations, number of kits mailed, list scrubbing, 12-month visit exclusion, reminder calls, patient incentive, and a mailed return option.

### Analysis

This study incorporated a multi-method approach. A descriptive analysis comparing CRC screening completion rates by health system characteristics and interventions was completed using Minitab and Tableau Software. Configurational Comparative Methods (CCMs) analyses were then performed using the R package “cna” to analyze the dataset using Coincidence Analysis (CNA) [29–31]. RStudio, R, and Microsoft Excel were also used to support the configurational analysis with CCMs. The configurational analysis examined the combinations of adaptations and health system characteristics that together distinguished the health systems with higher screening rates from those with lower screening rates.

The configurational analyses used a dichotomous outcome for each of three analyses: percent completed year 1, percent completed year 2, and change from year 1 to year 2 (positive or not positive). We set the threshold for our main outcome, CRC screening completion rate, at 19%. This cutoff was determined by tertiles, where we compared cases in the upper two tertiles of the screening rate percent versus cases in the lowest tertile. In the year 1 analysis, there were 17 cases in the overall sample, with 11 cases in the upper two tertiles and 6 cases in the lowest tertile. For year 2, there were 10 cases in the overall sample, with 7 cases in the upper two tertiles and 3 cases in the lowest tertile. In both year 1 and year 2, the 19% cut point separated the upper two tertiles from the lowest tertile, and in both years, there was a sizable performance gap in the outcome across this threshold, a difference of more than 3.5 points in absolute terms. Only health systems that participated in both years were included in the change from year 1 to year 2 analysis.

The configurational analyses produced an overall model with high consistency and coverage that identified combinations of conditions that explained the presence of the outcome. Consistency refers to how often health systems identified by the model had the outcome present (i.e., higher screening rates); coverage accounts for the percent of health systems with higher screening rates explained by the model.

To achieve data reduction, we used a configurational method to identify candidate factors, described in detail in prior studies [32–34]. To summarize, we used the “minimally sufficient conditions” function within the R package “cna” to look across all 17 cases and all 8 factors at once. The consistency threshold was initially set to 100% and the coverage threshold to 15%. We considered all 1-, 2-, 3-, 4-, and 5-condition configurations in our dataset that met this dual threshold. If no configurations met these criteria during the data reduction phase, we iteratively dropped the consistency threshold by increments of 5 percentage points (i.e., from 100 to 95%) and repeated the process of creating a new condition table until configurations emerged that satisfied all criteria.

Next, we sorted the condition table by complexity and coverage and identified the configurations with the highest coverage scores. We began with 1-condition configurations to see if they met the consistency and coverage thresholds and were uniquely distinguished from all other 1-condition configurations. We then proceeded to examine 2-, 3-, 4-, and 5-condition configurations, working upwards to minimize possible redundancy. Using this approach, we reduced the dataset to a smaller subset of candidate factors. We selected final solutions based on high overall model consistency (i.e., as close to 100% as possible, and at least 80%) and coverage (i.e., as close to 100% as possible, and at least 70%).

## Results

In total, 17 health systems (representing 51 total clinics) took part in at least 1 year of the BeneFIT program; 13 health systems were in the first year of the program and 14 were in the second year. Ten health systems took part in both program years. Table 1 shows implementation details by health system organized by decreasing rates of CRC screening in year 2 (2017) and FIT test type. Most health systems (12 of 17) used OC-Auto® or OC-Light® FIT tests. These health systems tended to be larger, with an average mailing size of 363 kits mailed and 3.4 clinics per system. The health systems that used “other types of FIT tests” tended to be smaller, with an average mailing size of 153 kits mailed and 2 clinics per system. Smaller health systems tended to use fewer adaptations. Small systems, defined to be one clinic, implemented fewer adaptations (mean = 1.75) than large systems (mean = 2.6) although the median number of adaptations was the same (median = 2).

**Table 1:** Participation details by health system and FIT test type

Health system	Health system (# of clinics)	2016 adaptations total (types)	2017 adaptations total (types)	2016 # mailed	2016 CRC screened total, (%)^a^	2017 # mailed	2017 CRC screened total (% of mailed)	Prior CRC research
**OC-Auto or OC-Light FIT**								
System 1	2	3 (M, I, E)	4 (M, Ph, S, E)	354	69 (19.5%)	194	68 (35.1%)	
System 2	5	N/A	3 (M, Ph, S)	N/A	N/A	630	198 (31.4%)	YES
System 3	2	3 (M, Ph, S)	3 (M, Ph, S)	111	25 (22.5%)	116	35 (30.2%)	
System 4	4	2 (M, Ph)	4 (M, Ph, S, E)	756	108 (14.3%)	379	104 (27.4%)	
System 5	5	2 (S, I)	2 (S, I)	326	86 (26.4%)	417	111 (26.6%)	YES
System 6	1	N/A	2 (M, Ph)	N/A	N/A	189	49 (25.9%)	
System 7	8	3 (M, S, I)	3 (M, S, I)	329	93 (28.3%)	354	87 (24.6%)	
System 8	7	1 (I)	3 (Ph, S, I)	628	124 (19.7%)	479	117 (24.4%)	YES
System 9	4	2 (M, Ph)	2 (M, Ph)	757	154 (20.3%)	626	152 (24.3%)	
System 10	1	1 (M)	3 (M, Ph, S)	132	20 (15.2%)	94	17 (18.1%)	
System 11	1	1 (M)	N/A	103	4 (3.9%)	N/A	N/A	
System 12	1	3 (M, Ph, S)	N/A	293	66 (22.5%)	N/A	N/A	
**Other FIT**								
System 13	1	N/A	1 (M)	N/A	N/A	105	20 (19.0%)	
System 14	2	2 (M, E)	2 (M, E)	57	14 (24.6%)	70	13 (18.6%)	
System 15	1	N/A	2 (M, E)	N/A	N/A	89	12 (13.5%)	
System 16	5	2 (M, Ph)	3 (M, Ph, S)	369	53 (14.4%)	343	42 (12.2%)	
System 17	1	1 (M)	N/A	44	4 (9.1%)	N/A	N/A	
**Overall**	**51 Clinics**	**--**	**--**	**4259**	**20%**	**4085**	**25%**	

*M* mailed versus in-person return, *Ph* phone reminders, *S* scrub list, *I* incentives, *E* 12-month exclusion

^a^CRC screened = claims submitted for any CRC screening procedure within 6 months of the introductory letter mailing date, CRC screened total number (% screened among those who received the intervention)

Table 2 shows yearly unadjusted CRC screening completion rates by adaptations and system characteristics. In 2016, the median completion rate was 20% among 13 health systems (range, 4–28%). In 2017, the median completion rate was 25% among 14 health systems (range, 12–35%). In both years, the median completion rates increased as the total number of adaptations increased. For the five interventions, scrubbing showed the largest difference in median rates for both years (15% vs 24% for no scrubbing vs scrubbing in 2016; 19% vs 27% for no scrubbing vs scrubbing for 2017). Median screening rates were higher in large systems, for OC-Auto® or OC-Light® FIT tests, and for systems with prior research study experience.

**Table 2:** Screening completion rates by adaptations and system characteristics

	2016		2017	
	Screening completion rate Median, % (range)	***N***	Screening completion rate Median, % (range)	***N***
**Rates overall**	**20% (4-28%)**	**13**	**25% (12-35%)**	**14**
**Rates by total number of adaptations**				
One	12% (4-20%)	4	19%	1
Two	20% (14-26%)	5	24% (13-27%)	5
Three	23% (19-28%)	4	25% (12-31%)	6
Four	*None*		31% (27-35%)	2
**Rates by individual adaptations**				
**Scrub lists**				
No scrubbing	15% (4-25%)	9	19% (13-26%)	5
Scrubbing	24% (23-28%)	4	27% (12-35%)	9
**Phone reminders**				
No phone reminders	20% (4-28%)	8	19% (13-27%)	5
Phone reminders	20% (14-23%)	5	26% (12-35%)	9
**Return by mail**				
No mailed return	23% (20-26%)	2	26% (24-27%)	2
Mail return allowed	19% (4-28%)	11	24% (12-35%)	12
**Incentives**				
No incentive	15% (4-25%)	9	24% (12-35%)	11
Incentives	23% (19-28%)	4	25% (24-27%)	3
**12-month exclusion**				
No exclusion	20% (4-28%)	11	25% (12-31%)	10
12-month exclusion	22% (19-25%)	2	23% (13-35%)	4
**Rates by system characteristics**				
**System size**				
Large (> 1 clinic)	20% (14-28%)	9	26% (12-35%)	10
Small	12% (4-23%)	4	19% (13-26%)	4
**Type of FIT**				
OC-Auto® or OC-Light®	20% (4-28%)	10	26% (18-35%)	10
Other	14% (9-25%)	3	16% (12-19%)	4
**Prior CRC study**				
None	19% (4-28%)	11	24% (12-35%)	11
Prior CRC study	23% (20-26%)	2	27% (24-31%)	3

*N* number of health systems

**Fig. 1:**
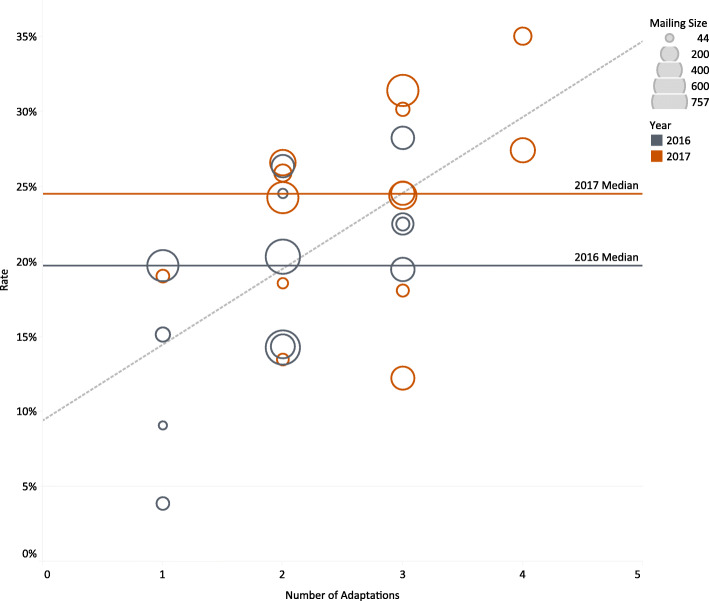
Completed screening rates by number of adaptations with year and mailing size. *Mailing size is the number of kits mailed for each system and is shown by the size of the circles in the plot. Mailing size spanned a low of 44 to a high of 757 kits

Figure 1 presents a multivariate visualization indicating that screening completion rates were positively associated with the number of adaptations implemented by a health system. Note that all systems tried at least one adaptation, and no system tried all five. The size of each mailing is also indicated in the plot, and systems with larger mailings tended to implement more adaptations. Systems with smaller mailings used fewer adaptations and had lower screening completion rates. To characterize the general relationship between screening completion rates and number of adaptations, we fitted a weighted regression line, with weights determined by the number of mailed kits. The slope of the weighted regression line was 0.04, suggesting that, on average, the screening rate increased by 4% for each additional adaptation (*P* = 0.006). Figure 1 also shows that the screening rates were generally higher in the second year of the study.

Health system size was not sufficient by itself to account consistently for implementation outcomes. In year 1, for example, while larger systems on average did tend to have more implementation success, three of the eleven systems with implementation success were small (only one clinic); moreover, two of the six systems without implementation success in year 1 were in the larger size category (≥ 2 clinics). In year 2, the system with the lowest screening rate overall (12.2.%) was in the largest category of health system size (≥ 5 clinics).

Combinations of conditions were formally assessed in the second phase of analysis using the configurational approach. In the year 1 model, 11 of the 17 health systems had screening rates over 19%, i.e., had a successful outcome as defined by the model, while 6 did not. The final model for year 1 featured four solution pathways (i.e., four different ways to achieve the outcome). The model had a consistency level of 100% (9/9) and a coverage level of 82% (9/11) (i.e., it explained nine of 11 health systems with the outcome present). Table 3 lists health systems with the explanatory factors (adaptations or clinic characteristics) that contributed to the solution pathways; the highlighted cells show the combinations that led to a successful outcome. While health system size was not sufficient by itself to explain implementation outcomes, health system size played a pivotal role in the configurational solutions, as health system size in conjunction with other specific conditions consistently distinguished systems with implementation success. Small health systems (with only one clinic) that used phone reminders represented one solution pathway for higher rates of CRC completion (*n* = 2). Health systems with more than one clinic, by contrast, were successful if they offered a gift card incentive (*n* = 4), scrubbed the lists prior to mailing (*n* = 4), or allowed mailed versus in-person return but had no other adaptations (*n* = 1). In the year 1 dataset, two systems had a contradictory configuration, meaning they had identical values for all potential explanatory factors but different outcomes and thus could not be explained by the factors in the analysis.

**Table 3:**
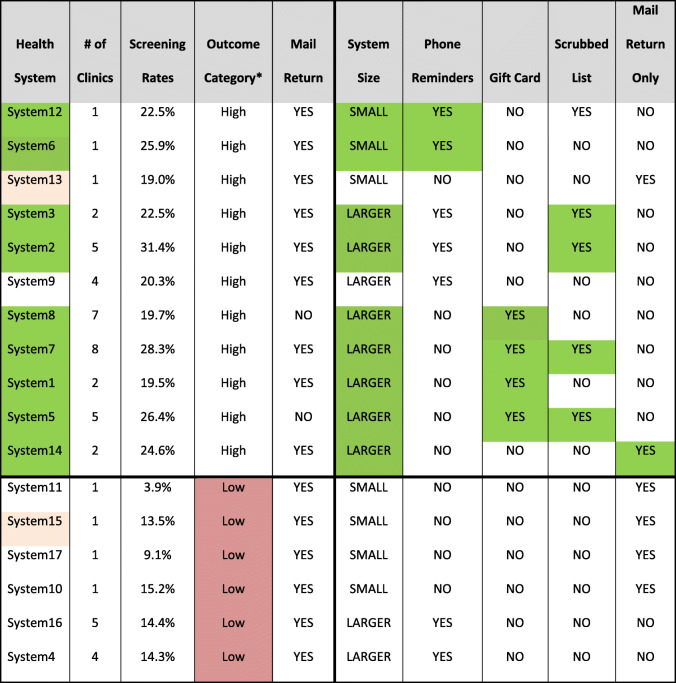
Year 1 CCMs model with four solution pathways

The highlighted cells (above the black dividing line) show the solution combinations that led to a successful outcome

*Outcome category High is considered a CRC screening rate ≥ 19% for the purpose of this analysis

Green shading indicates cases with higher screening rates covered by at least one solution pathway

Salmon shading indicates cases with a different outcome but the same adaptations

The year 2 configurational analysis (data not shown) included ten health systems that participated in both years of the study. In year 2, seven of the 10 health systems had the outcome present, while three did not. The final year 2 model featured two solution pathways with no inconsistent cases: (1) offering a gift card incentive (*n* = 2) or (2) a phone reminder conducted by a health system size of 2-5 clinics (*n* = 4). The model had a consistency level of 100% and a coverage level of 86% (i.e., it explained six of seven systems with higher rates with perfect consistency). FIT type did not show up in any of the solution pathways.

### Comparison of first and second year CRC screening completion rates

Of the 10 health systems that took part in both years of the program, three systems had lower rates in year 2 than year 1, whereas the remaining seven had positive gains in year 2 over year 1.

The third configurational analysis assessed the change in screening rates from year 1 to year 2 in the 10 systems that participated in both years of the program (Table 4). The results yielded three solutions with 100% consistency (5/5) and 71% coverage (5/7). Higher rates of change were found in health systems that implemented phone reminders (*n* = 3) in year 2 after not offering them in year 1, that instituted a 12-month visit requirement in year 2 (*n* = 1) after not requiring it in year 1, or that had participated in the prior research study (*n* = 2).

**Table 4:**
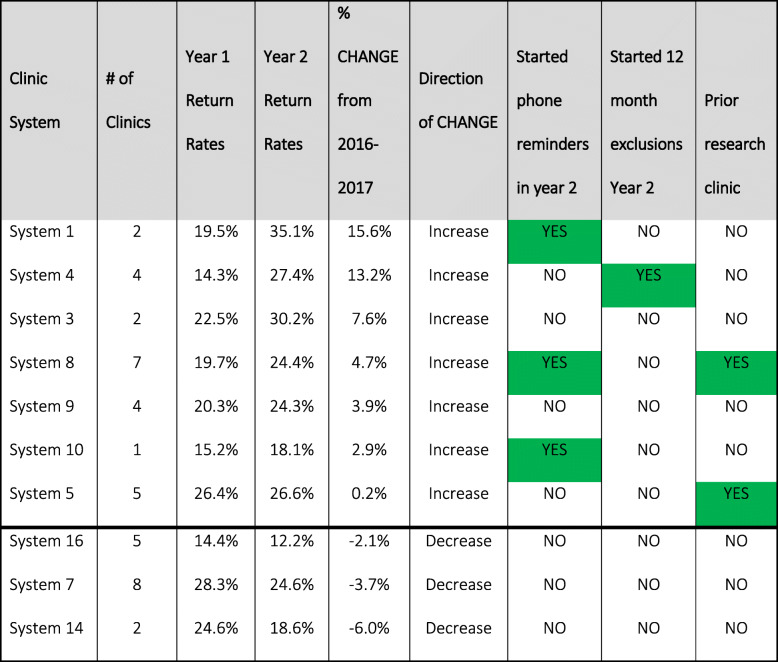
CRC screening rate change from years 1 to 2, among health systems in both years (*n* = 10)

## Discussion

Our multi-method analysis did not find a single adaptation that improved response rates across all clinics. While no single adaptation was the “secret sauce” for implementation success, our linear regression results do indicate that the centralized mailed FIT program was more effective when multiple adaptations were added to the health plan’s basic program. Health systems achieved higher rates when they were able to combine the health plan’s program with two or more of the following adaptations: a phone call reminder, reviewing the mailing lists (e.g., scrubbing), allowing mailed FIT returns, excluding patients without a recent visit, or offering financial incentives. This finding that more comprehensive efforts yielded higher screening rates supports prior research suggesting that FIT return rates can be increased by delivering more and additional types of reminders [35–37].

The configurational analyses identified multiple ways for health systems to achieve higher rates of screening. It found size to be an important contextual factor, with different solutions for larger health systems than those for smaller health systems. Phone call reminders appeared in multiple solutions, consistent with findings from other studies [27]. Systems that added phone calls in year 2, instituted a 12-month visit requirement in year 2, or had been in prior research studies achieved higher screening rates in year 2 over year 1, indicating that a focus on specific process improvements can lead to success.

A comparative analysis of the STOP CRC study, the intervention upon which the BeneFIT program was based, found two conditions that accounted for successful implementation (i.e., percent of eligible patients mailed a FIT): having a centralized process for delivering the intervention and mailing an introductory letter prior to the FIT [38]. These two implementation components indicated a greater clinic capacity to staff the program and internal commitment to the evidence-based research. The BeneFIT study had a similar mailed FIT program, but in a centralized capacity with a vendor mailing all components. Therefore, mailing implementation was no longer an issue, and we were able to look at the effect of health system factors and adaptations on screening completion rates. The variation in BeneFIT results might indicate differing health system commitment to the program or capacity to add additional implementation components. However, while the screening rate was an average of 4 percentage points higher for each additional adaptation, we found different combinations leading to a successful overall result. Therefore, while it is tempting to conclude the “secret sauce” is more adaptations, we need to consider *which* adaptations are effectively combined. A key benefit of configurational analyses is to help understand which adaptations result in the greatest impact so that low-resource clinics can choose options that are most effective.

Screening rates generally improved over time for the health systems that took part in both years; other literature supports this finding [39, 40]. Staff and patients becoming familiar with the FIT screening process, consistent messaging with patients, and conversations between patients and physicians might have contributed to higher screening rates. Health systems that had participated in the prior STOP CRC research had higher screening rates in year 2, perhaps indicating that they already had staff and workflow familiarity for a mailed FIT program. Baker et al.’s patient-level study of FIT mailing with phone call and text message reminders suggested that prior FIT screening might be a predictor of screening completion [41]. Therefore, those patients screened in year 1 might have been more likely to complete the FITs in year 2 leading to higher system screening rates.

These results have several limitations. We used observational data, with no control group of health systems for comparison. Also, our sample size was not large enough to stratify the FIT test brands (or one-sample vs. two-sample FIT tests) into different groups. Our data analysis is based on claims submitted to the health plan; therefore, we cannot ascertain if health systems became more efficient at submitting claims in year 2. Finally, we could not conclude that there was a direct effect of individual adaptations on outcomes because the health system size and FIT test type variables were confounded with adaptations. Some health systems used multiple adaptations but still had low screening rates that did not improve. These results were possibly related to factors we could not measure, such as populations that are more resistant to the mailed FITs, lab or mailing issues, or FIT processing issues. Some adaptations (such as phone call reminders) could have been variably implemented. Also, this outreach was offered in addition to existing in-clinic screening efforts (such as direct provider outreach) that we do not capture here.

Despite these limitations, these results can offer guidance to health systems on implementation efforts. FIT is a lower barrier method towards achieving greater CRC screening rates, which is a metric used by several national agencies such as UDS reporting, HEDIS measures, and Medicare STARS metrics.

## Conclusions

Overall, our results identified several solution paths to implementation of a successful mailed FIT program. Larger and smaller health systems may be able to use different approaches to adapting an outreach intervention offered by their health plan. If a health plan can be flexible in its approach, it could benefit from customizing the approach to CRC screening outreach to particular environments or clinics. Future research might help establish the strength of the causal relationships between specific conditions and CRC screening rates.

## Data Availability

The datasets used and/or analyzed for the current study are available from the corresponding author on reasonable request. Templates used for the mailed FIT program materials and implementation workflow are available at the mailedfit.org website.

## Abbreviations

CRC: Colorectal cancer
FIT: Fecal immunochemical testing
CCMs: Configurational comparative methods
EHR: Electronic health record
FOBT: Fecal occult blood test
CT: Computed tomography colonoscopy
CNA: Coincidence analysis
